# Microbial Community and Potential Pathogen Shifts Along an Ornamental Fish Supply Chain

**DOI:** 10.3390/microorganisms6030091

**Published:** 2018-08-25

**Authors:** Linda A. Amaral-Zettler, Victor Schmidt, Katherine F. Smith

**Affiliations:** 1 The Josephine Bay Paul Center for Comparative Molecular Biology and Evolution, Marine Biological Laboratory, 7 MBL Street, Woods Hole, MA 02543, USA; linda.amaral-zettler@nioz.nl (L.A.A.-Z.); victor.schmidt@tuebingen.mpg.de (V.S.); 2 Department of Earth, Environmental and Planetary Sciences, Brown University, Providence, RI 02912, USA; 3 Department of Marine Microbiology and Biogeochemistry, NIOZ Royal Netherlands Institute for Sea Research and Utrecht University, P.O. Box 59, 1790 AB Den Burg, The Netherlands; 4 Department of Ecology and Evolutionary Biology, Brown University, 80 Waterman Street Box G-W, Providence, RI 02912, USA; 5 Max Planck Institute for Developmental Biology, Department of Microbiome Sciences, 72076 Tübingen, Germany

**Keywords:** amplicon sequencing, Sailfin Tang, fish microbiome

## Abstract

There is broad interest in disease spread through the pet trade, but empirical research on hosts and pathogens in transit along actual trade routes is notably absent. Using next-generation DNA sequencing, and partnering with the ornamental fish industry, we tracked shifts in microbial community and potential pathogen structure associated with Sailfin Tang (*Zebrasoma desjardinii*) along the United States (U.S.) leg of an international supply chain. We observed striking changes in microbial diversity and composition of potential pathogens, including increased dominance of vibrios of fishes in transit. Our pilot findings suggest that high investment in fishes early in the supply chain may not matter to their long-term health depending on end destination conditions.

## 1. Introduction

Between 2000–2005, over half a million shipments containing >1.4 billion live wildlife animals entered the United States (U.S.), 99% intended for sale in the pet industry [1]. Pathogens of wild animals are spread through contact between susceptible and infected individuals that depends on animal behavior, pathogen prevalence, and host population dynamics. However, within supply chains, pathogen load should depend more so on industry choices of how animals are handled, housed, and transported. Even under the best conditions, animals in transit are subject to chronic stress that can alter their microbiomes, including the relative abundance of opportunistic pathogens, and increased susceptibility to disease [2,3]. Despite growing interest in disease spread through pet trade, empirical research on host-pathogen dynamics along actual trade routes has been almost non-existent (however, see [3]). This is largely due to proprietary barriers and industry wariness that prevent direct access to animals in trade. We had the novel opportunity to overcome this hurdle by capitalizing on established partnerships with two professional businesses in the ornamental fish industry.

Ornamental fishes are the third most popular pet in U.S. homes with ownership increasing more than 20% over the past decade [4]. We have identified significant microbial diversity and potential pathogens in tank water housing ornamental fishes in U.S. aquarium/pet shops [5], but microbiome changes as animals move through the supply chain remains unexplored. In 2010/2011 we worked with a Los Angeles-based marine ornamental fish importer and wholesale distributor, and a pet shop on Cape Cod in Massachusetts that sells ornamental fishes, to conduct a pilot survey of the microbial communities associated with fishes and their carriage water along one leg of an international supply chain. The L.A. distributor is a 35-year old business with state-of-the-art marine fish holding facilities and a leader in post-import acclimation investment, filtration capacity, and system stability. The Massachusetts business is representative of most pet/aquarium shops selling ornamental marine fishes in the U.S., offering ~10–20 species originating in Florida, the Caribbean and Southeast Asia, and utilizing a combination of multi-tank filtration systems and individual aquarium charcoal based filters. Both businesses remain anonymous for this report.

## 2. Materials and Methods

We purchased three Sailfin Tang (*Zebrasoma desjardinii*), among the most popular marine species imported to and sold in the U.S. pet trade (unpublished data from [1,5]) directly from the L.A. distributor and sampled the water that the fish would be shipped in prior to departure from the L.A. facility. The fish were wild caught in Indonesia and shipped directly to the L.A. facility. After four days of post-import acclimation, the distributor shipped bags to the Massachusetts shop with and without fish (three replicates each) so we could compare the taxonomic composition and relative abundance of bacteria (the microbiome) of the water alone, to that of the water with fish added. The fish were shipped separately in individual bags, a standard practice. Approximately 20 h after initial packing/sampling in L.A, the fish arrived at the Massachusetts shop where we immediately sampled bag water with and without fish, as well as fish mucus. Upon arrival, each of the fish appeared healthy and active. The fish were then added to shop tanks already containing other fishes and invertebrates (~7 species/20 individuals) and cared for by shop employees, using standard practices, for two weeks. At one and two week intervals, we collected one liter of tank water for microbiome investigation. In week two, we sacrificed the fish to examine the resulting fish-associated microbiome. To further explore the possibility of moribund fish microbiomes contributing to pathogen loads in pet store tanks, we examined the microbiomes of moribund freshwater and marine fishes randomly collected from the Massachusetts pet shop to identify potential pathogens in the community. Pet shop employees collected fishes within ~60 min of morbidity and froze them until we were able to process them.

Fish were euthanized in MS-222. Mucus samples were collected via sterile cotton swabs run laterally along the length of the fish. Mucus samples were placed in 1× Phosphate Buffered Saline (PBS) and frozen at −20 °C. Tissue was homogenized in 20–40 mL of 0.22 µm-filtered 1× PBS using sterilized scissors (MBL IACUC 11-27, Woburn, MA, USA). Homogenate was passed through a 5-µm filter followed by centrifugation for 10 min at 10,000× *g* to remove eukaryotic cells. Genomic DNA was extracted from the resulting bacterial pellets using the Puregene Yeast/Bacteria kit (Qiagen, Valencia, CA, USA) following the manufacturer’s protocol and DNA was purified using PowerDNA Cleanup kit (MoBio, Carlsbad, CA, USA). Bacterial V6–V4 hypervariable regions of the 16S rRNA gene were amplified using primers targeting *E. coli* positions 518 and 1046 (Table S1) and sequenced on a 454 Genome Sequencer FLX (Roche, Basel, Switzerland) using the GS-FLX–Titanium platform and multiplexing strategy [6]. Sequences of adapters and primers (Table S1) were trimmed, and low-quality and chimeric sequences were removed as described previously [7]. Three percent Operational Taxonomic Units (OTUs) were assigned using the UCLUST v3.0.617 de novo clustering algorithm [8]. Global Alignment Sequence Taxonomy (GAST) algorithms assigned taxonomy to the most abundant read within an OTU by querying databases of known bacterial taxonomies as described previously [9]. OTU bar graphs were generated using GAST taxonomy and graphical output in QIIME v1.2.0 [10]. MIMARKS-compliant data (Table S2) are deposited in NCBI’s Sequence Read Archive (SRP016028) (Table S3) [11].

## 3. Results and Discussion

**Figure 1:**
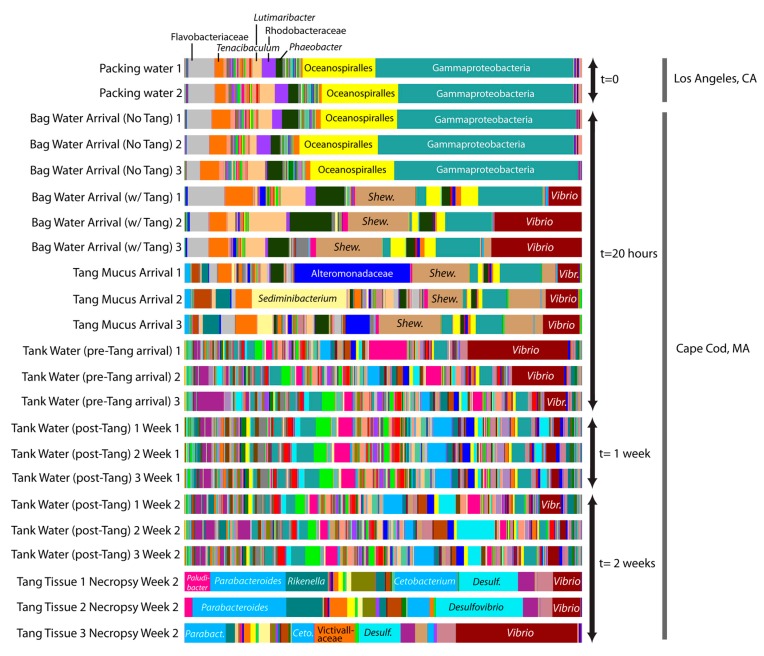
Bar graphs depicting relative changes in Operational Taxonomic Unit (OTU) composition at the genus level based on V6–V4 rRNA gene amplicon sequencing of samples associated with the shipment of Sailfin Tang (*Zebrasoma desjardinii*) collected along a U.S. leg of an international ornamental fish supply chain (Los Angeles distributor’s facility to Massachusetts pet shop). *Vibrio* genera are highlighted and labeled in red. *Shewanella* genera are highlighted and labeled in tan. Other dominant taxa are indicated as shown. Please see Figure S1 for a complete legend of OTU-assigned taxonomy.

We detected major changes in the microbial communities associated with *Z. desjardinii* transport from L.A. to Massachusetts (Figure 1). Packing water communities from the L.A. distributor and shipment water in bags containing no Tang (sampled upon arrival at the Massachusetts shop) were very similar. The microbial community of shipment water containing the Tang differed substantially from shipment water without fish (Figure 1) over the supply chain and seemed responsible for elevated levels of *Vibrio* and *Shewanella* species in particular (Figure 1). The water the Tang were placed into before shipping showed <0.03% *Vibrio* species while addition of the Tang increased the vibrios to 14.9% (±4.1) of the bacterial population and 19% in the highest replicate (after 20 h).

The tank water in the Massachusetts shop had greater microbial richness and evenness (Shannon index: 9.84 (±0.95), *n* = 3) compared to the Tang themselves (Shannon index: 5.30 (±0.55), *n* = 3) or shipment water (Shannon index: 5.02 (±1.25), *n* = 3) (Figure 1). Bacterial communities awaiting the fish in the Massachusetts shop’s tanks consisted of 4.6% (±3.5%) *Vibrio* genera prior to Tang introductions. These vibrios were highly diverse and included OTUs related to *Vibrio* spp., *V. aestuarianus, V. agarivorans, V. alginolyticus, V. azureus, V. campbellii, V. cholerae, V. fortis, V. harveyi, V. metschnikovii, V. parahaemolyticus, V. sinaloensis,* and *V. vulnificus*. Of these, *V. alginolyticus* and *V. vulnificus* are known fish pathogens. An OTU related to *Shewanella* spp. was also found at greater abundance in the “Bag Water Arrival with Tang” added sample (14% ± 0.01) versus controls or tank water (0%). This OTU was also found in high abundance in fish mucus (11% ± 0.01) and fish tissue (0.9% ± 0.005). Species of *Shewanella* have been implicated in food-fish spoilage [12].

**Figure 2:**
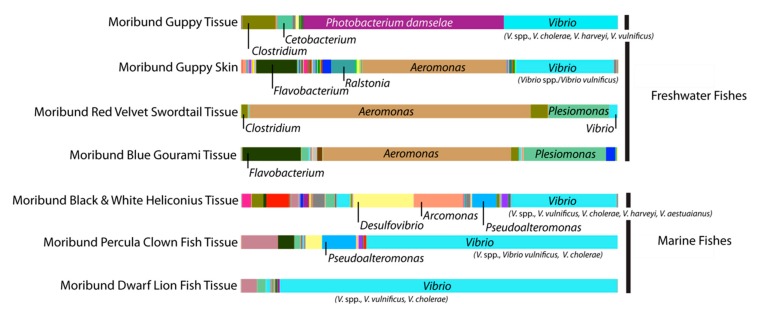
Bar graphs of bacterial Operational Taxonomic Units (OTUs) at the genus level based on V6–V4 rRNA gene amplicon sequencing from moribund freshwater and marine fish microbiomes from a Massachusetts pet shop. Marine species displayed larger relative abundances of *Vibrio* genera, while freshwater species showed dominance by *Photobacterium* or *Aeromonas*. All three genera include species potentially pathogenic to fishes. The V6–V4 hypervariable region cannot differentiate between certain species of vibrios, hence some of the vibrios are designated spp.

Our pilot study revealed significant changes in microbial diversity, relative abundance, and composition of potential pathogens (i.e., *Vibrio* and *Shewanella* spp.) associated with a popular marine ornamental fish at three sampling points along the U.S. leg of an international supply chain. In this pilot study we were unable to collect samples associated with the Tang before they entered the U.S., otherwise we would have been able to ascertain differences between the fish microbiomes prior to arrival versus after arrival at the pet shop. Our data also identified a potentially important aspect of supply chains wherein seemingly healthy fish that arrive at the end of the trade route (e.g., at a pet/aquarium shop) may become infected with potential pathogens already in tanks awaiting them. To explore this, we further examined the microbiomes of moribund freshwater and marine fishes randomly collected from the Massachusetts pet shop to identify potential pathogens in the community (Figure 2).

The genus *Aeromonas* dominated the moribund freshwater fish microbiome along with the genus *Plesiomonas* to a lesser extent (Figure 2). Both of these genera include species that are potential fish pathogens. In contrast, moribund marine fishes had increased levels of vibrios associated with them (Figure 2). *Vibrio vulnificus* appears to be the likely fish pathogen common to the three different marine fish species sampled. However, we cannot rule out the possibility of multiple vibrio infections, or some other factor, being the cause of morbidity as pathological investigation was beyond the scope of this project. *Aeromonas* and *Vibrio* species are common inhabitants of healthy ornamental fishes and aquatic systems that can become pathogenic and cause 100% mortality when conditions are stressful [13,14]. The release of pathogenic bacteria from morbid and dead fishes into carriage/tank water shared by other animals can exacerbate the risk of disease in these systems and can result in large economic losses to industry [15]. Water quality is one of the most important contributors to fish health and stress level [16] and our pilot survey reveals how dramatically it can vary between hubs of an ornamental fish supply chain. Stressors, like poor water quality, encountered at the end of the supply chain can be especially detrimental as fishes are likely to be in a state of chronic stress by this stage and subsequently more vulnerable to disease, morbidity, and mortality. Losses of fish in the ornamental trade have been noted to be greatest within several weeks of arrival at pet/aquarium shops (industry partners, personal communication).

Sixty-two percent of U.S. households own a pet, the majority more than one, supporting a >$50 billion industry annually [4]. Pet ownership provides broad economic and social benefits, and so it is realistic to expect that the wildlife trade to support the pet industry will continue to thrive and that some disease risks will always exist for animals, industry handlers, and owners. Our pilot study demonstrates a potential way to reduce this risk—a win-win situation where working directly with reputable industry professionals can identify supply chain hubs and conditions detrimental to the health of animals in trade. Stress may be an inevitable component of pet trade, but identifying ways to reduce the timing and impact of chronic stress should increase survivorship and reduce exposure to pathogens shed by diseased animals. We recommend science-industry partnerships to reduce potential pathogens along supply chains and protect pets and their owners.

## Supplementary Materials

The following are available online at http://www.mdpi.com/2076-2607/6/3/91/s1, Table S1: Description of 454-specific adaptor sequences, and our universal bacteria V6–V4 16S rRNA gene primer sequences used to generate sequenced amplicons (see vamps.mbl.edu), Table S2: MIMARKS-compliant table of sample associated meta-data. Table S3: Excel file depicting the relative percentage of a given taxonomic group to species level within each sample. Data are available for visualization using the tools available at http://vamps.mbl.edu and logging in as guest. Figure S1: Legend to accompany Figure 1 summarizing the taxonomic composition for each sample. OTU tables were generated using a hybrid USEARCH [8], GAST [9] pipeline, and graphics were generated in Qiime v 1.2.0 [10].

## References

[1] Smith K.F., Behrens M., Schloegel L.M., Marano N., Burgiel S., Daszak P. (2009). Reducing the risks of the wildlife trade. Science.

[2] Dickens M.J., Delehanty D.J., Romero L.M. (2010). Stress: An inevitable component of animal translocation. Biol. Conserv..

[3] Smith K.F., Yabsley M.J., Sanchez S., Casey C.L., Behrens M.D., Hernandez S.M. (2012). *Salmonella* isolates from wild-caught Tokay geckos (*Gekko gecko*) imported to the US from Indonesia. Vector Borne Zoonotic Dis..

[4] 2011-2012 APPA National Pet Owners Survey. https://faunalytics.org/2011-2012-appa-national-pet-owners-survey/.

[5] Smith K.F., Behrens M.D., Max L.M., Daszak P. (2008). US drowning in unidentified fishes: Scope, implications, and regulation of live fish import. Conserv. Lett..

[6] Huber J.A., Mark Welch D.B., Morrison H.G., Huse S.M., Neal P.R., Butterfield D.A., Sogin M.L. (2007). Microbial population structures in the deep marine biosphere. Science.

[7] Huse S.M., Huber J.A., Morrison H.G., Sogin M.L., Mark Welch D. (2007). Accuracy and quality of massively parallel DNA pyrosequencing. Genome Biol..

[8] Edgar R.C. (2010). Search and clustering orders of magnitude faster than BLAST. Bioinformatics.

[9] Huse S.M., Dethlefsen L., Huber J.A., Mark Welch D., Relman D.A., Sogin M.L. (2008). Exploring microbial diversity and taxonomy using SSU rRNA hypervariable tag sequencing. PLoS Genet..

[10] Caporaso J.G., Kuczynski J., Stombaugh J., Bittinger K., Bushman F.D., Costello E.K., Fierer N., Pena A.G., Goodrich J.K., Gordon J.I. (2010). QIIME allows analysis of high-throughput community sequencing data. Nat. Methods.

[11] Yilmaz P., Kottmann R., Field D., Knight R., Cole J.A., Amaral-Zettler L.A., Gilbert J.A., Karsch-Mizrachi I., Johnston A., Cochrane G. (2011). The “Minimum Information about a MARKer gene Sequence” (MIMARKS) specification. Nat. Biotechnol..

[12] Gram L., Dalgaard P. (2002). Fish spoilage bacteria—Problems and solutions. Curr. Opin. Biotechnol..

[13] Francis-Floyd R. *Aeromonas* Infections. http://ufdc.ufl.edu/IR00001327/00001.

[14] Reed P.A., Francis-Floyd R. *Vibrio* Infections of Fish. http://www.aces.edu/dept/fisheries/education/ras/publications/Update/Vibrio%20infections%20in%20fish.pdf.

[15] Neuhaus H., Meyer K. (2006). Koi herpesvirus infection of carp (*Cyprinus carpio* L.). Kleintierpraxis.

[16] Shelton J.L., Roberts H.E. (2010). Water quality. Fundamentals of Ornamental Fish Health.

