# Supplementary material for: Microbial Community and Potential Pathogen Shifts Along an Ornamental Fish Supply Chain
Source: Microorganisms. 2018 Aug 25;6(3):91. doi: 10.3390/microorganisms6030091 (PMC6164381; doi:10.3390/microorganisms6030091)
Supplement: Supplementary file 1 [file microorganisms-06-00091-s001.zip › TableS1_primers.docx]

**Table S1.** Description of 454-specific adaptor sequences, and our universal bacteria V6-V4 16S rRNA gene primer sequences used to generate sequenced amplicons (see vamps.mbl.edu).

| **454 adaptor Sequences** |  |  |  |
| --- | --- | --- | --- |
| Roche A-adaptor 5'-GCCTCCCTCGCGCCATCAG-3' | | |  |
| Roche B-adaptor 5'-GCCTTGCCAGCCCGCTCAG-3' | | |  |
| **Primer Sequences** |  |  |  |
| Forward primer (518F): CCAGCAGCYGCGGTAAN | | |  |
| Reverse primer 1 (1046R-1) CGACRRCCATGCANCACCT | | |  |
| Reverse primer 2 (1046R-2) CGACAGCCATGCANCACCT | | | |
| Reverse primer 3 (1046R-3) CGACAACCATGCANCACCT | | | |
| Reverse primer 4 (1046R-4) CGACGGCCATGCANCACCT | | | |
| Reverse primer 5 (1046R-5) CGACGACCATGCANCACCT | | | |
